# Carbon-Source Dependent Interplay of Copper and Manganese Ions Modulates the Morphology and Itaconic Acid Production in *Aspergillus terreus*

**DOI:** 10.3389/fmicb.2021.680420

**Published:** 2021-05-20

**Authors:** Erzsébet Sándor, István S. Kolláth, Erzsébet Fekete, Vivien Bíró, Michel Flipphi, Béla Kovács, Christian P. Kubicek, Levente Karaffa

**Affiliations:** ^1^Institute of Food Science, Faculty of Agricultural and Food Science and Environmental Management, University of Debrecen, Debrecen, Hungary; ^2^Department of Biochemical Engineering, Faculty of Science and Technology, University of Debrecen, Debrecen, Hungary; ^3^Doctoral School of Chemistry, University of Debrecen, Debrecen, Hungary; ^4^Juhász-Nagy Pál Doctoral School of Biology and Environmental Sciences, University of Debrecen, Debrecen, Hungary; ^5^Institute of Chemical, Environmental & Bioscience Engineering, TU Wien, Vienna, Austria

**Keywords:** *Aspergillus terreus*, manganese(II) ions, copper(II) ions, itaconic acid overflow, pentose, hexose, mycelial pellets, divalent cation antagonism

## Abstract

The effects of the interplay of copper(II) and manganese(II) ions on growth, morphology and itaconic acid formation was investigated in a high-producing strain of *Aspergillus terreus* (NRRL1960), using carbon sources metabolized either mainly via glycolysis (D-glucose, D-fructose) or primarily via the pentose phosphate shunt (D-xylose, L-arabinose). Limiting Mn^2+^ concentration in the culture broth is indispensable to obtain high itaconic acid yields, while in the presence of higher Mn^2+^ concentrations yield decreases and biomass formation is favored. However, this low yield in the presence of high Mn^2+^ ion concentrations can be mitigated by increasing the Cu^2+^ concentration in the medium when D-glucose or D-fructose is the growth substrate, whereas this effect was at best modest during growth on D-xylose or L-arabinose. *A. terreus* displays a high tolerance to Cu^2+^ which decreased when Mn^2+^ availability became increasingly limiting. Under such conditions biomass formation on D-glucose or D-fructose could be sustained at concentrations up to 250 mg L^–1^ Cu^2+^, while on D-xylose- or L-arabinose biomass formation was completely inhibited at 100 mg L^–1^. High (>75%) specific molar itaconic acid yields always coincided with an “overflow-associated” morphology, characterized by small compact pellets (<250 μm diameter) and short chains of “yeast-like” cells that exhibit increased diameters relative to the elongated cells in growing filamentous hyphae. At low concentrations (≤1 mg L^–1^) of Cu^2+^ ions, manganese deficiency did not prevent filamentous growth. Mycelial- and cellular morphology progressively transformed into the typical overflow-associated one when external Cu^2+^ concentrations increased, irrespective of the available Mn^2+^. Our results indicate that copper ions are relevant for overflow metabolism and should be considered when optimizing itaconic acid fermentation in *A. terreu*s.

## Introduction

Itaconic acid (2-methylenesuccinic acid, 1-propene-2,3-dicarboxylic acid) is an unsaturated, weak diprotic acid. Its distinguished chemical properties are related to the conjugated double bond of the methylene group that allows polymerization both by addition and condensation, as well as the esterification of the carboxylic groups with different co-monomers (Kuenz et al., 2012). Itaconic acid is used as a building block for a wide variety of industrial products of added value such as resins, paints, synthetic fibers, plasticizers, and detergents (Okabe et al., 2009). Itaconic acid applications have also penetrated the dental, ophthalmic and drug delivery fields (Hajian and Yusoff, 2015). A promising chemical for the bio-based economy, itaconic acid polymers may even substitute petroleum-based polyacrylic acid, presenting a potential multi-billion dollar market (Cunha da Cruz et al., 2018).

On technical scale, itaconic acid is produced by batch fermentation in a process largely similar to that of citric acid, employing the ascomycete filamentous fungus *Aspergillus terreus*. The highest published specific molar yield of itaconic acid is about 85% of the theoretical value, accounting to a volumetric yield of ∼150 g L^–1^ (Karaffa et al., 2015). Itaconic acid overflow requires an excess of rapidly metabolizable carbon source, high dissolved oxygen levels, an initial medium pH between 3 and 5, a phosphate concentration low enough to limit fungal growth, and a paucity of manganese ions (Mn^2+^) (Hevekerl et al., 2014; Molnár et al., 2018; Karaffa and Kubicek, 2019; Saha and Kennedy, 2019b). In *Aspergilli*, manganese deficiency transforms filamentous hyphal morphology to one dominated by “yeast-like cells” (branches of short, swollen forms) on the micro-morphology-, and small compact (<0.5 mm diameter) pellets on the macro-morphology level (Detroy and Ciegler, 1971; Gyamerah, 1995). Progression of this transformation is accompanied by increased cell/hyphae diameter and reduced pellet size (Paul et al., 1994; Karaffa et al., 1997; El-Sabbagh et al., 2008). High (>75%) specific molar itaconic acid yields (*Y*_*p/s*_) occur only when cultures overwhelmingly comprise such morphology (henceforth referred to as “overflow-associated morphology”), improving the rheology of the culture broth, which in turn results in increased oxygen transfer throughout the fermentation (Kuenz et al., 2012; Karaffa et al., 2015).

Excess of copper(II) (Cu^2+^) ions results in mitigation of the deleterious effect that Mn^2+^ ions exert on the formation of citric acid in *A. niger*, possibly by interfering with cellular Mn^2+^ ion uptake and -homeostasis (Hockertz et al., 1987; Netik et al., 1997). It has recently been demonstrated that the Cu^2+^ ion concentration is a significant process variable for *A. terreus* itaconic acid fermentations, an attribute to further product yield in the presence of inhibitory concentrations of Mn^2+^ ions (Saha and Kennedy, 2019b). These antagonistic properties allowed the formulation of synthetic media for improved production of itaconic acid without the necessity to pretreat the carbon source with cation exchange resins to remove associated Mn^2+^. Industrial itaconic acid fermentations mostly utilize molasses or starch hydrolysates as the source of carbon (for a review, see Karaffa and Kubicek, 2019). However, competition with food applications increasingly impels the industry to utilize sugars released from non-food, lignocellulosic plant biomass (Cunha da Cruz et al., 2018; Kuenz and Krull, 2018). Lignocellulose is a complex network of carbohydrate polymers of predominantly hexose or pentose monomers crosslinked by lignin, highly inert polymers containing phenolic and other aromatic subunits (Saha, 2003; Saha et al., 2017). However, release, biodegradation, uptake and metabolic conversion of the different polysaccharide components and their constituent monosaccharides cross interact and interfere with one another. It is thus crucial to understand their individual metabolism and bioconversion first to appreciate their individual contributions to sustainable production of itaconic acid from cheap raw vegetative material (Kolláth et al., 2019).

The ability of Cu^2+^ ions to alleviate the negative effect of Mn^2+^ ions on citric acid and itaconic acid fermentations have almost exclusively been investigated on hexoses. The objective of this study was to investigate whether itaconic acid production on other monosaccharide carbon sources that constitute lignocellulose shows similar responses to the co-presence of Cu^2+^ and Mn^2+^. We investigated the influence of Cu^2+^ ions on the growth, morphology and itaconic acid formation in the high-titer itaconic acid producer *Aspergillus terreus* NRRL1960 on D-glucose, D-xylose, L-arabinose and an alternative glycolytic carbon source, D-fructose. We show that on D-glucose and D-fructose, the ratio of Cu^2+^ to Mn^2+^ – rather than their respective absolute concentrations – co-determines micro- and macromorphology of the biomass and modulates the itaconic acid production yield in batch cultures. However, on D-xylose and L-arabinose the collaborative antagonism between the two divalent cations is less straightforward, taking into account the increased sensitivity of *A. terreus* to Cu^2+^ of when growing on either of these two pentose sugars.

## Materials and Methods

### Fungal Strain and Cultivation Conditions

*Aspergillus terreus* NRRL 1960 (CBS 116.46; ATCC 10020), a standard high-producer strain, was maintained on agar plates (Kuenz et al., 2012). Per liter of distilled water, the chemically defined minimal medium used throughout the experiments contained 0.1 g KH_2_PO_4_, 3 g NH_4_NO_3_, 1 g MgSO_4_ × 7 H_2_O, 5 g CaCl_2_ × 2 H_2_O, 1.67 mg FeCl_3_ × 6 H_2_O, 8 mg ZnSO_4_ × 7 H_2_O. The default concentration for Cu^2+^ ions – in the form of CuSO_4_ × 7 H_2_O – was 3.3 mg L^–1^ (cf. Kuenz et al., 2012), but it was set between 0.01 and 1000 mg L^–1^ in the diverse experiments. Hexoses D-glucose and D-fructose and pentoses D-xylose and L-arabinose were used as sole carbon sources. Initial concentrations were 50 g L^–1^ for D-xylose (Kolláth et al., 2019), 80 g L^–1^ for L-arabinose (Saha et al., 2017), and 120 g L^–1^ for D-glucose (Karaffa et al., 2015) and D-fructose (data not shown; concentration optimized during preliminary experiments). Fermentations were carried out either under manganese(II) ion limitation (=1.5 μg L^–1^) or sufficiency (=300 μg L^–1^). To control the concentration of Mn^2+^ ions in the growth medium, all carbon sources were dissolved in distilled water and passed through a column (440 × 45 mm) of Dowex 50 W-X8 (100/200) cation exchange resin. All other medium components were added to these sugar solutions from sterile stock solutions made up with Dowex 50 W-X8 treated water. The final Mn^2+^ concentration in the medium was adjusted by the addition of appropriate volumes of a stock solution of MnCl_2_ × 4H_2_O.

Shake-flask cultivations were performed in 500-mL Erlenmeyer flasks (VWR International Kft., Debrecen, Hungary) with 100 mL medium incubated at 33°C in a rotary shaker (Infors AG, Basel, Switzerland) operating at 300 revolutions per minute (rpm). This aeration regime is optimal for the production of itaconic acid in shake flasks, regardless of the growth substrate (Karaffa et al., 2015). The initial medium pH was set at 3.0 with 3 M HCl, and was not controlled during the fermentations (Karaffa et al., 2015).

Bioreactor cultivations were carried out in 2.5-L glass fermentors (Sartorius AG, Göttingen, Germany) with a culture (working) volume of 2 L, equipped with one six-blade Rushton disc turbine impeller. Operating conditions were 33°C, and 0.75 vessel volume per minute (vvm) of aeration. Before inoculation, the pH was adjusted to 3.0 with 3 M HCl but was not controlled during fermentation. Dissolved oxygen (DO) levels were maintained at 30% saturation by adjusting the impeller tip speed. DO, temperature, and impeller tip speed were controlled automatically by the regulatory units of the bioreactors. To minimize water loss, the waste gas (from the headspace) was cooled in a reflux condenser connected to an external cooling bath (4°C) before exiting the system. Both shake-flask and bioreactor cultures were inoculated with 1 × 10^6^ *A. terreus* conidia per milliliter of medium from a freshly prepared, high-density spore suspension in a 1/10,000 Tween-20 solution.

All chemicals used were of analytical grade and purchased from Sigma-Aldrich.

### Analytical Methods

Mycelial dry cell weight (DCW) was determined from 5-mL culture aliquots as described by Kozma and Karaffa (1996). The biomass was harvested on a pre-weighted glass wool filter and washed with cold tap water, after which the filter was dried at 70°C until constant weight. Biomass yields (*Y*_*x/s*_) were calculated by dividing the concentration of the highest biomass (DCW) value by that of the total supplied carbon source. Specific growth rates (μ, given as the reciprocal of time, h^–1^) were calculated from the DCW increase over the time elapsed between two consecutive sampling time points; the highest of the thus obtained values was taken as the maximal specific growth rate of the culture.

The concentrations of the sugar carbon sources and itaconic acid in the growth media were determined by high-pressure/performance liquid chromatography (HPLC; Agilent 1260 Infinity II with Quaternary pump and Vial sampler) with a proton exchange column (Bio-Rad Aminex HPX-87H^+^) at 55°C, using isocratic elution with 10 mM H_2_SO_4_ and refractive index detection as described by Fekete et al. (2002). The concentrations were calculated from two independent measurements, which never deviated more than 5%. Specific molar itaconic acid yield (*Y*_*p/s*_) is the ratio of the produced moles of itaconic acid and the consumed moles of carbon source. Biomass-specific itaconic acid yield (*Y*_*p/x*_) is the ratio of the volumetric yield of itaconic acid (g L^–1^) and the DCW measured at the same time-point.

Manganese- and copper ion concentrations in the growth media were determined by inductively coupled plasma quadrupole mass spectrometry (ICP-QMS; Thermo Fisher Scientific, Bremen, Germany) equipped with Hexapole Collision Cell Technology (CCT), as described in detail by Karaffa et al. (2015).

Copper toxicity was defined as the external Cu^2+^ concentration in the culture broth at which half-maximal specific growth rate values (LD_50_) were attained under the given growth conditions. The monitored effect of the toxic compound was the de-acceleration of growth during cultivation, assessed with successive DCW determinations. This approach is illustrated by Supplementary Figure 1, which shows the maximum specific growth rate versus the Cu^2+^ concentration on D-glucose both at low- and high manganese concentrations.

Fungal morphology was defined in three forms – (a) the swollen hyphal fragments called yeast-like forms, (b) the filamentous hyphae and (c) the mycelial pellets that are spherical colonies of highly entangled hyphal biomass (Bartoshevich et al., 1990; Paul and Thomas, 1998). Morphology was investigated microscopically with an Axio-Vision AC quantitative image analyzer system. To increase contrast and visibility, lactophenol cotton blue (Fluka Chemie, Buch, Switzerland) was added to the medium samples in a final concentration of 10 % (v/v), except when spore germination and agglutination was monitored. Stained samples were analyzed with a Zeiss AxioImager phase-contrast microscope, equipped with AxioCam MRc5 camera. Average cell- and average pellet diameters (also referred to as micro- and macro-morphology, respectively) were assessed with the AxioVision AC image analyzer system processing at least 50 cells or 10 pellets for each liquid culture sample studied.

### Reproducibility

All presented data are the means of three to five independent experiments (biological replicates: starting with liquid cultures using different spore inocula). Data were analyzed and visualized with Sigmaplot software (Jandel Scientific), and for all datasets standard deviations were determined. Quantitative data (*n* ≥ 3) were compared using ANOVA (Analysis of Variance) with the Holm-Sidak-Test for pairwise comparisons. While probability (*p*) values were often <0.001, the criterion for significance was *p* < 0.05 in all cases.

## Results

### Verification of the Experimental System

A high number of growth conditions (cultures) was implicated to study the interplay of the two essential divalent cations and the consequences for itaconic acid overflow. Therefore shake-flasks were initially the cultivation vehicle of choice. To substantiate our findings toward application in scaled-up fermentations, we also performed controlled batch cultivations in 2-L scale bioreactors for those cultivation regimes that appeared crucial to verify and demonstrate the principles of our findings and their future exploitation. To ensure that the major physical parameters (pH, DO, temperature) of the two cultivation systems were essentially identical, and thus that fermentation kinetics would be independent of the vessel type, protocols for monitoring pH and DO were applied as previously described by us (Kolláth et al., 2019). Although either peak values of fungal biomass- and itaconic acid formation, as well as the residual carbon concentrations of the cultures grown in shake-flask or bioreactor deviated sometimes up to 20% at a given time point, the trends were always consistent. We thus considered our experimental setup appropriate for the purposes of this study.

### Copper(II) Ion Tolerance of *Aspergillus terreus* Depends on the Carbon Source and the Concentration of Manganese Ions

A synthetic growth medium optimized for itaconic acid production (Kuenz et al., 2012) was used to test copper tolerance of *A. terreus* NRRL1960 using two hexoses (D-glucose, D-fructose) and two pentoses (D-xylose, L-arabinose) as carbon sources. Each carbon source was used at a concentration that allowed the highest itaconic acid yield, i.e., 120 g L^–1^ for D-glucose and D-fructose, 80 g L^–1^ for L-arabinose and 50 g L^–1^ for D-xylose (see “Materials and Methods” for details). The default Cu^2+^ concentration that allowed the highest growth rate was 3.3 mg L^–1^ for each carbon source tested. The Mn^2+^ ion concentration was set either at 1.5 μg L^–1^ – which is growth limiting but favors itaconic acid production – or 300 μg L^–1^, which is optimal for biomass formation (Saha and Kennedy, 2019a).

In the presence of 300 μg L^–1^ manganese ions, the addition of increased concentrations of copper ions started to decrease the biomass yield from the carbon source (*Y*_*x/s*_) at concentrations >50 mg L^–1^ (with the exception of L-arabinose where the decrease was only apparent at >100 mg L^–1^). Yet the half-maximal lethal concentrations (LD_50_) of copper ions on the two hexoses were higher (close to 1 g L^–1^) than on the two pentoses (around 0.75 g L^–1^) (Tables 1A, 2A). In the presence of 1.5 μg L^–1^ manganese ions, however, the decrease in biomass yield started already at copper concentrations of >75 mg L^–1^ on the two hexoses and at >25 mg L^–1^ on the two pentoses (Table 2B). In agreement with these findings, the half-maximal lethal concentration of copper ions was around 100 mg L^–1^ for the two hexoses, and 78 mg L^–1^ for D-xylose. The LD_50_ value of Cu^2+^ for L-arabinose was even only 48 mg L^–1^ (Table 1A). The sensitivity of *A. terreus* to Cu^2+^ ions thus appears to depend on the concentration of manganese ions as well as on the growth substrate, particularly at limiting concentrations of Mn^2+^.

**TABLE 1A:** Copper(II) ion toxicity of *A. terreus* NRRL1960 grown on four different carbon sources in liquid minimal media either under manganese(II) ion limitation (1.5 μg L^–1^) or under manganese(II) ion sufficiency (300 μg L^–1^).

	D-glucose	D-fructose	D-xylose	L-arabinose
[300 μg L^–1^ Mn]	981 mg L^–1^	948 mg L^–1^	752 mg L^–1^	734 mg L^–1^
[1.5 μg L^–1^ Mn]	105 mg L^–1^	102 mg L^–1^	78 mg L^–1^	48 mg L^–1^

The germination of conidiospores tolerated much higher concentrations of copper – up to 3 g L^–1^ for hexoses in the presence of 300 μg L^–1^ manganese ions (Table 1B) – but otherwise showed the same trend as the biomass yield *Y*_*x/s*_, the sensitivity being higher at limiting manganese concentrations and during growth on pentoses (Table 1B). Interestingly, the determined LD_50_ values did not depend on the concentration of the carbon source as we obtained essentially the same threshold values for conidiospore germination on all four carbon sources tested across a range of 10–150 g L^–1^ initial carbon source concentration (data not shown).

**TABLE 1B:** 

	D-glucose	D-fructose	D-xylose	L-arabinose
[300 μg L^–1^ Mn]	3 000 mg L^–1^	3 000 mg L^–1^	2 000 mg L^–1^	2 000 mg L^–1^
[1.5 μg L^–1^ Mn]	250 mg L^–1^	250 mg L^–1^	100 mg L^–1^	100 mg L^–1^

*All other nutrients, including the carbon source, were present at default concentrations (see “Materials and Methods” section). Growth media were inoculated with a freshly prepared conidiospore suspension.*

***(A)** LD50 values, i.e., the calculated copper(II) concentrations causing half of the maximal specific growth rate (h^–1^) measured at the optimal copper(II) concentration (3.3 mg L^–1^).*

***(B)** Lowest copper(II) ion concentration at which germination of conidiospores could not be observed.*

### The Concentrations of Cu^2+^ and Mn^2+^ Influence the Itaconic Acid Yield in a Carbon Source-Dependent Manner

**FIGURE 1:**
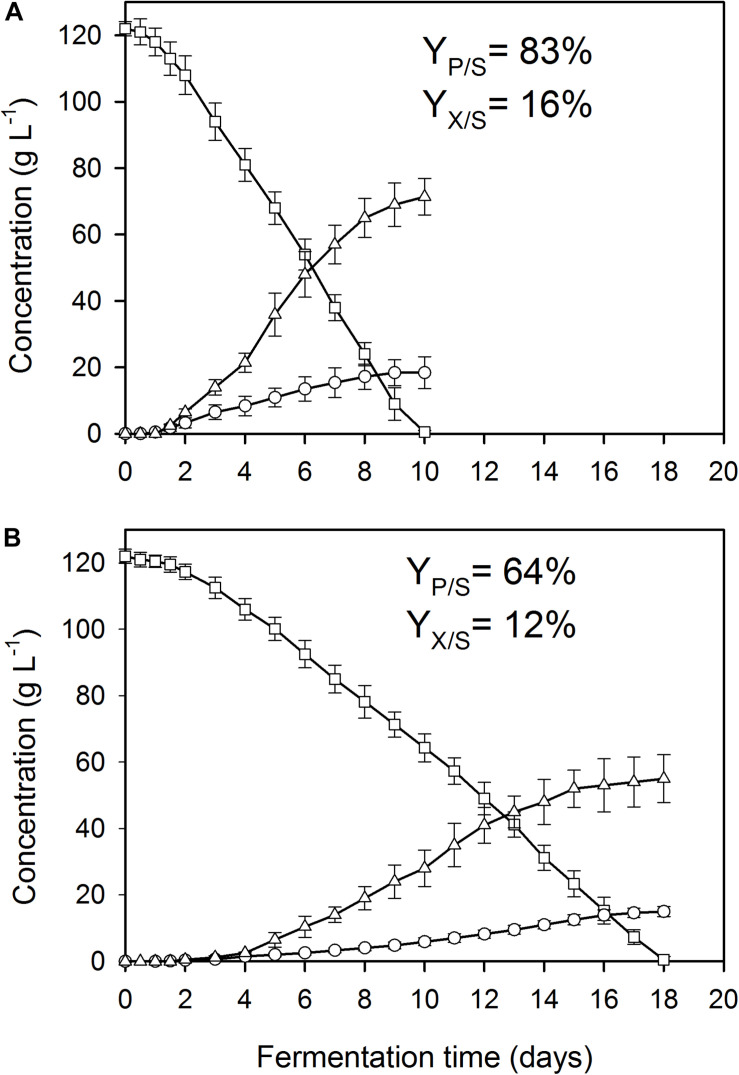
Kinetics of fungal biomass (O), itaconic acid (△), and residual D-glucose (□) concentrations in controlled batch fermentations of *A. terreus* NRRL 1960, grown in a 2-L scale bioreactor in the presence of 3 μg L^–1^ Mn^2+^ ions (manganese limitation). The variables share the same *y*-axis as they are expressed in the same unit (g L^–1^). *Y*_*p/s*_, molar itaconic acid yield; *Y*_*x/s*_, biomass (DCW) yield. **(A)** Culture supplemented with 3.3 mg L^–1^ Cu^2+^ ions. **(B)** Culture supplemented with 0.01 mg L^–1^ Cu^2+^ ions.

Under fully optimized fermentation conditions (initial substrate concentration, culture broth pH, Mn^2+^ limitation and high DO) *A. terreus* NRRL1960 is capable of converting >80% of the available D-glucose, on a molar basis (*Y*_*p/s*_), into itaconic acid (Karaffa et al., 2015). Slightly lower specific yields were achieved on D-fructose, whereas significantly lower yields were attained on D-xylose and particularly on L-arabinose (Table 2A). Specific molar itaconic acid yields were always highest at a Cu^2+^ concentration of 3.3 mg L^–1^. On the two hexoses, the lowest yields were observed at the lowest of the Cu^2+^ concentrations tested (0.01 mg L^–1^; Figure 1B). On D-glucose, D-fructose and D-xylose, the molar itaconic acid yield *Y*_*p/s*_ decreased only slightly at copper concentrations higher than 3.3 mg L^–1^, which was accompanied by an increased yield of itaconic acid per biomass unit (*Y*_*p/x*_). However, unlike hexose-grown cultures, the pentose-grown cultures did not form any itaconic acid at Cu^2+^ concentration of ≥75 mg L^–1^ (Table 2A). Finally, the generally low itaconic acid yields on L-arabinose did not seem to variate with the copper concentration in the growth medium.

**TABLE 2A:** Specific molar itaconic acid yield (*Y*_*p/s*_), biomass-specific itaconic acid yield (*Y*_*p/x*_) and biomass yield (*Y*_*x/s*_) of *Aspergillus terreus* NRRL1960 cultures as a function of the copper(II) ion concentration in the culture broth.

1.5 μg L^–^^1^ Mn^2+^												

	D-glucose			D-fructose			D-xylose			L-arabinose		
				
Cu^2+^ (mg L^–1^)	*Y*_*x/s*_	*Y*_*p/s*_	*Y*_*p/x*_	*Y*_*x/s*_	*Y*_*p/s*_	*Y*_*p/x*_	*Y*_*x/s*_	*Y*_*p/s*_	*Y*_*p/x*_	*Y*_*x/s*_	*Y*_*p/s*_	*Y*_*p/x*_
**0.01**	**0.12 ± 0.01**	**0.64 ± 0.02**	**3.60 ± 0.4**	0.10 ± 0.01	0.57 ± 0.01	3.40 ± 0.3	0.10 ± 0.01	0.46 ± 0.03	3.0 ± 0.4	0.10 ± 0.01	0.24 ± 0.05	3.20 ± 0.4
1	0.14 ± 0.01	0.70 ± 0.03	3.80 ± 0.4	0.12 ± 0.02	0.77 ± 0.03	3.60 ± 0.4	0.12 ± 0.01	0.56 ± 0.02	3.80 ± 0.4	0.11 ± 0.01	0.32 ± 0.05	3.30 ± 0.5
**3.3**	**0.16 ± 0.01**	**0.83 ± 0.01**	**3.90 ± 0.5**	0.15 ± 0.02	0.80 ± 0.03	3.80 ± 0.3	0.15 ± 0.01	0.58 ± 0.03	4.90 ± 0.5	0.14 ± 0.03	0.32 ± 0.03	4.20 ± 0.4
10	0.15 ± 0.03	0.80 ± 0.03	3.90 ± 0.4	0.15 ± 0.01	0.77 ± 0.01	3.80 ± 0.4	0.15 ± 0.01	0.45 ± 0.05	4.40 ± 0.3	0.13 ± 0.01	0.32 ± 0.01	4.80 ± 0.5
25	0.15 ± 0.05	0.81 ± 0.05	3.80 ± 0.5	0.15 ± 0.02	0.76 ± 0.02	3.80 ± 0.3	0.11 ± 0.02	0.41 ± 0.04	4.80 ± 1.6	0.11 ± 0.01	0.29 ± 0.01	5.0 ± 2.4
50	0.15 ± 0.01	0.79 ± 0.01	3.50 ± 0.3	0.14 ± 0.03	0.76 ± 0.03	3.50 ± 0.4	0.08 ± 0.01	0.38 ± 0.04	7.10 ± 2.3	0.05 ± 0.01	0.30 ± 0.01	7.90 ± 3.3
75	0.11 ± 0.03	0.81 ± 0.03	4.40 ± 1.5	0.12 ± 0.02	0.78 ± 0.02	4.30 ± 1.4	0.04 ± 0.01	No IA	No IA	0.04 ± 0.01	No IA	No IA
100	0.05 ± 0.02	0.78 ± 0.02	8.0 ± 1.9	0.04 ± 0.01	0.74 ± 0.01	8.20 ± 1.8	No growth	No growth	No growth	No growth	No growth	No growth
250	0.03 ± 0.02	0.70 ± 0.03	12.50 ± 2.7	0.02 ± 0.01	0.68 ± 0.03	12.10 ± 4.3	No growth	No growth	No growth	No growth	No growth	No growth
300	No growth	No growth	No growth	No growth	No growth	No growth	No growth	No growth	No growth	No growth	No growth	No growth
400	No growth	No growth	No growth	No growth	No growth	No growth	No growth	No growth	No growth	No growth	No growth	No growth

### Mn^2+^ Inhibition of Itaconic Acid Formation Is Mitigated by Cu^2+^ Ions in a Carbon Source-Dependent Manner

**FIGURE 2:**
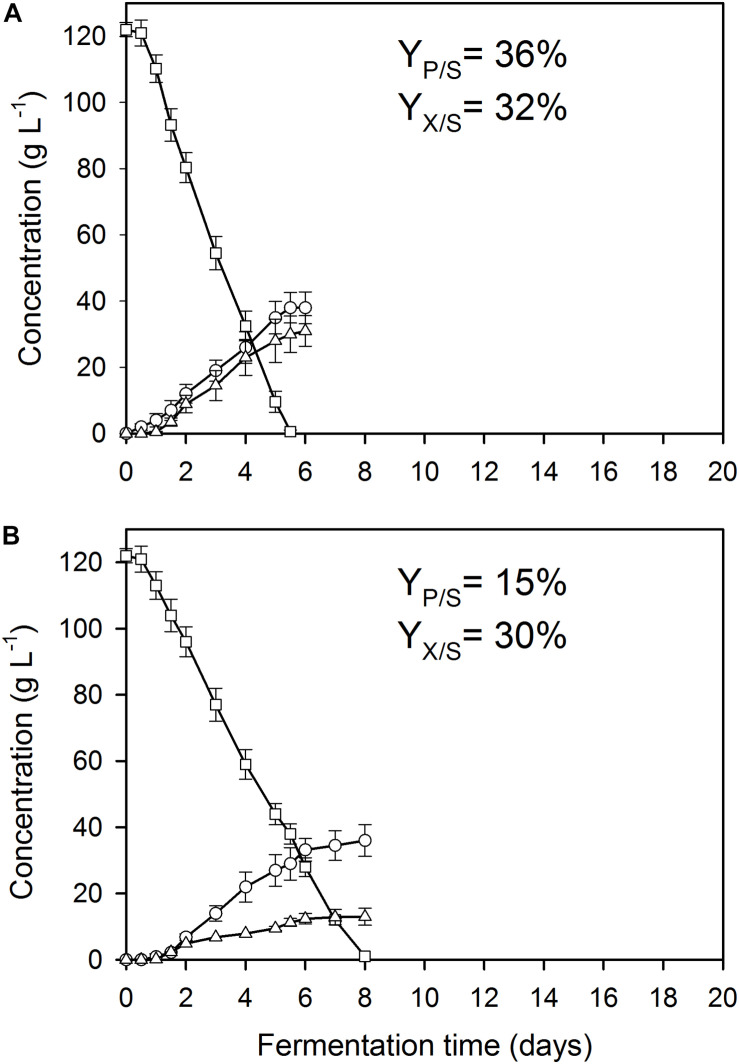
Kinetics of fungal biomass (O), itaconic acid (△), and residual D-glucose (□) concentrations in controlled batch fermentations of *A. terreus* NRRL 1960, grown in a 2-L scale bioreactor in the presence of 300 μg L^–1^ Mn^2+^ ions. The variables share the same *y*-axis as they are expressed in the same unit (g L^–1^). **(A)** Culture supplemented with 3.3 mg L^–1^ Cu^2+^ ions. **(B)** Culture supplemented with 0.01 mg L^–1^ Cu^2+^ ions.

**FIGURE 3:**
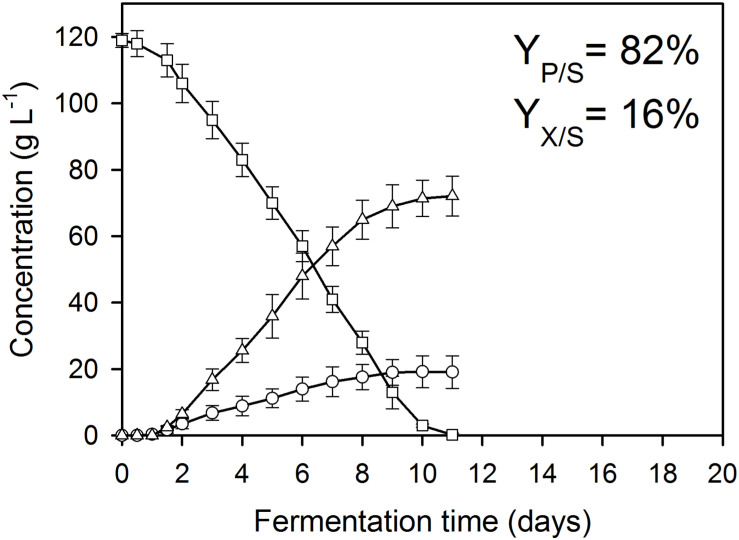
Kinetics of fungal biomass (O), itaconic acid (△), and residual D-glucose (□) concentrations in controlled batch fermentations of *A. terreus* NRRL 1960, grown in a 2-L scale bioreactor in the presence of 300 μg L^–1^ Mn^2+^ and 300 mg L^–1^ Cu^2+^ ions. The variables share the same *y*-axis as they are expressed in the same unit (g L^–1^).

As reported previously for D-glucose and D-xylose (Karaffa et al., 2015; Kolláth et al., 2019), increasing the extracellular Mn^2+^ ion concentrations in the medium (to 300 μg L^–1^ in this study) significantly lowered the maximal *Y*_*p/s*_ of itaconic acid on all four carbon sources. At 3.3 mg L^–1^ Cu^2+^, i.e., the concentration conducing optimal itaconic acid production under conditions of Mn^2+^ paucity, and 300 μg L^–1^ Mn^2+^, the molar yield decreased by 21% on D-glucose (Figure 2A) and by 16% on D-fructose relative to the best conditions for itaconic acid production (Table 2A). Lowering the Cu^2+^ ion concentration down to 0.01 mg L^–1^ did not change biomass production of the cultures but significantly decreased molar itaconic acid yield (Figure 2B). However, increasing the copper(II) ion concentration within the same range of concentrations as in the earlier experiments with Mn^2+^ ion limited cultivations described above, gradually increased the specific molar itaconic acid yield. On D-glucose, essentially the same itaconic acid yields could be reached at 1.5 μg L^–1^ Mn^2+^/3.3 mg L^–1^ Cu^2+^ (Figure 1A) and at 300 μg L^–1^ Mn^2+^/300 mg L^–1^ Cu^2+^ (Figure 3). Similarly, molar yields could be restored to over 90% of the optimal yield on D-fructose by increasing the amount of Cu^2+^ ions by two orders of magnitude to counteract the 200-fold excess of Mn^2+^. However, contrary to the situation on the two glycolytic hexoses, the inhibitory effect of manganese(II) ion sufficiency on itaconic acid production was not fully alleviated by an excess of copper(II) ions in the case of either of the pentoses as the growth substrate. On D-xylose, *Y*_*p/s*_ did increase with rising copper ion concentrations, but only to half of the maximal yield obtained at 1.5 μg L^–1^ Mn^2+^, whereas no attenuation could be observed on L-arabinose as the carbon source (Tables 2A,B).

**TABLE 2B:** 

300 μg L^–^^1^ Mn^2+^												

	D-glucose			D-fructose			D-xylose			L-ARABINOSE		
				
Cu^2+^ (mg L^–1^)	*Y*_*x/s*_	*Y*_*p/s*_	*Y*_*p/x*_	*Y*_*x/s*_	*Y*_*p/s*_	*Y*_*p/x*_	*Y*_*x/s*_	*Y*_*p/s*_	*Y*_*p/x*_	*Y*_*x/s*_	*Y*_*p/s*_	*Y*_*p/x*_
0.01	**0.30 ± 0.03**	**0.15 ± 0.03**	**0.36 ± 0.1**	0.28 ± 0.04	0.12 ± 0.04	0.30 ± 0.2	0.25 ± 0.02	0.06 ± 0.02	0.2 ± 0.1	0.25 ± 0.03	No IA	No IA
1	0.31 ± 0.03	0.31 ± 0.03	0.75 ± 0.1	0.29 ± 0.01	0.29 ± 0.01	0.70 ± 0.3	0.29 ± 0.02	0.06 ± 0.02	1.1 ± 0.2	0.30 ± 0.04	0.02 ± 0.04	0.6 ± 0.2
3.3	**0.32 ± 0.04**	**0.36 ± 0.06**	**0.81 ± 0.1**	0.33 ± 0.02	0.34 ± 0.02	0.75 ± 0.3	0.30 ± 0.02	0.09 ± 0.02	1.4 ± 0.2	0.28 ± 0.04	0.03 ± 0.05	1.2 ± 0.3
10	0.31 ± 0.03	0.40 ± 0.03	1.1 ± 0.2	0.33 ± 0.01	0.43 ± 0.01	0.95 ± 0.4	0.30 ± 0.04	0.11 ± 0.04	1.6 ± 0.4	0.29 ± 0.03	0.03 ± 0.06	1.9 ± 0.1
25	0.30 ± 0.03	0.51 ± 0.03	1.8 ± 0.3	0.30 ± 0.02	0.48 ± 0.02	1.5 ± 0.5	0.31 ± 0.01	0.17 ± 0.01	1.9 ± 0.3	0.29 ± 0.04	0.03 ± 0.06	0.5 ± 0.1
50	0.25 ± 0.03	0.65 ± 0.03	2.5 ± 0.3	0.24 ± 0.03	0.66 ± 0.03	2.4 ± 0.3	0.28 ± 0.02	0.22 ± 0.02	2.5 ± 0.5	0.29 ± 0.04	No IA	No IA
75	0.22 ± 0.03	0.67 ± 0.06	2.5 ± 0.3	0.20 ± 0.02	0.67 ± 0.02	2.3 ± 0.2	0.27 ± 0.01	0.24 ± 0.01	1.2 ± 0.3	0.29 ± 0.04	No IA	No IA
100	0.19 ± 0.03	0.65 ± 0.03	2.6 ± 0.3	0.20 ± 0.02	0.66 ± 0.02	2.7 ± 0.2	0.25 ± 0.03	0.25 ± 0.03	0.3 ± 0.1	0.23 ± 0.04	No IA	No IA
250	0.17 ± 0.03	0.73 ± 0.04	3.1 ± 0.4	0.16 ± 0.03	0.68 ± 0.03	3.5 ± 0.3	0.19 ± 0.03	No IA	No IA	0.20 ± 0.04	No IA	No IA
300	**0.16 ± 0.01**	**0.82 ± 0.01**	**3.8. ± 0.5**	0.15 ± 0.01	0.71 ± 0.01	3.6 ± 0.5	0.17 ± 0.03	No IA	No IA	0.16 ± 0.04	No IA	No IA
400	0.14 ± 0.03	0.79 ± 0.03	3.5. ± 0.3	0.13 ± 0.03	0.72 ± 0.03	3.5 ± 0.5	0.13 ± 0.03	No IA	No IA	0.12 ± 0.04	No IA	No IA

*Shake flask cultures were grown on four different carbon sources either under manganese(II) ion limitation (1.5 μg L^–1^);*

***(A)** or under manganese(II) ion sufficiency (300 μg L^–1^);*

***(B)**. All other nutrients, including the carbon source, were present at default concentrations (see “Materials and Methods” section). The D-glucose fermentations of which the respective yield constants are highlighted in bold were also performed in 2-L scale bioreactors for verification of the shake flask results (see “Results” section). IA, itaconic acid.*

### The Ratio of Manganese and Copper Ions Concentrations Affects Fungal Morphology

Under conditions conductive to itaconic acid production, the morphology of D-glucose-grown *A. terreus* cultures is characterized by small, compact pellets and yeast-like cells (Supplementary Figure 2), rather than elongated hyphae (Karaffa et al., 2015). They are characterized by increases in cell diameter and decreases in pellet diameters. These two measurable parameters were therefore assessed for variation during the course of the fermentations. On D-glucose at 1.5 μg L^–1^ Mn^2+^ ions, the average cell diameter in the 24-h old cultures was 2.41 ± 0.58 μm when 0.01 mg L^–1^ Cu^2+^ ions were present in the culture broth, and displayed a continuous increase up until a maximum diameter was observed at 25 mg L^–1^ (Table 3A). Above 25–50 mg L^–1^ Cu^2+^ the trend reversed, and the average cell diameter were not significantly lower (3.57 ± 1.04) at 250 mg L^–1^ Cu^2+^ than they were at 0.01 mg L^–1^. The difference between the most extreme cell diameters observed was over four-fold, and the largest cell diameters were observed in the cultures with the highest molar itaconic acid yields. These trends were similar at each time-point tested, from a day after inoculation until carbon source exhaustion, although the span between the two most extreme cell diameter values decreased with the culture age. Pellet sizes followed an opposite pattern in that the pellets were at their largest at 0.01 mg L^–1^ Cu^2+^, and sharply decreased in diameter with increasing Cu^2+^ concentrations. The pellets were at their smallest at 50 mg L^–1^ Cu^2+^ (Table 4A).

**TABLE 3A:** Average cell diameter in *A. terreus* NRRL1960 D-glucose-grown mycelia as a function of the copper(II) ion concentration in the culture broth at Mn(II) limitation and sufficiency.

	1.5 μg L^–^^1^ Mn^2+^										

Cu^2+^ (mg L^–1^)	0.01	1	3.3	10	25	50	75	100	250	300	400

*Mn:Cu* × *10*^3^	*150*	*1.5*	*0.45*	*0.15*	*0.06*	*0.03*	*0.02*	*0.015*	*0.006*	*0.005*	*0.00375*
24 h	2.41 ± 0.58	3.88 ± 1.25	7.76 ± 1.84	9.59 ± 1.41	10.90 ± 1.84	9.36 ± 1.95	7.76 ± 1.31	6.59 ± 1.19	3.57 ± 1.04	N.G.	N.G.
48 h	3.52 ± 1.08	4.01 ± 1.47	6.93 ± 1.55	9.75 ± 0.97	11.49 ± 1.56	11.47 ± 1.43	7.84 ± 1.10	5.15 ± 1.28	4.05 ± 1.43	N.G.	N.G.
72 h	3.56 ± 0.89	4.66 ± 1.78	7.56 ± 1.93	8.16 ± 2.13	10.76 ± 4.17	10.40 ± 1.78	7.21 ± 2.20	4.89 ± 1.90	6.59 ± 1.58	N.G.	N.G.
96 h	3.95 ± 1.04	4.46 ± 1.88	7.88 ± 2.29	9.15 ± 2.00	12.77 ± 1.98	12.68 ± 1.92	8.08 ± 2.18	4.71 ± 2.24	5.63 ± 1.43	N.G.	N.G.
168 h	3.90 ± 1.18	4.77 ± 1.67	8.08 ± 1.36	9.23 ± 1.38	13.77 ± 2.60	10.26 ± 2.09	9.61 ± 1.58	6.94 ± 2.28	5.86 ± 1.73	N.G.	N.G.

Fungal morphology was fundamentally different in the presence of sufficient Mn^2+^ ions in the growth medium, 300 μg L^–1^. Maximal average cell diameters from early time-point samples were either smaller or similar than the later ones, and the diameters were generally lower than those measured under Mn^2+^ limitation (Table 3B). Cultures with Cu^2+^ ion concentrations in the range of 0.01 and 100 mg L^–1^ displayed mostly filamentous morphology with average cell diameters less than 2.5 μm. Cultures with copper concentrations above 100 mg L^–1^ had increasingly higher cell diameters, particularly in the later stages of cultivation (72, 96, 168 h). At 300 and 400 mg L^–1^ Cu^2+^ ions, average cell diameters were 60–70% of the values measured in cultures grown under itaconic acid production conditions (Table 3B). Our data suggest that variation in average cell diameter is correlated with the ratio of manganese(II) and copper(II) ions in the fermentation rather than with the concentration of either of the two cations. Hyphal diameter remained ∼ 2 μm during the course of the fermentation as long as the Mn:Cu ratio was higher than 1.2 × 10^–3^. However, average diameter of cells – as well as specific molar itaconic acid yields – significantly increased when the Mn:Cu ratio fell between 1 × 10^–3^ and 0.75 × 10^–3^. No such correlation was found in the cultures grown at low Mn^2+^ ion concentrations.

**TABLE 3B:** 

	300 μg L^–^^1^ Mn^2+^										

Cu^2+^ (mg L^–1^)	0.01	1	3.3	10	25	50	75	100	250	300	400

*Mn:Cu* × *10*^3^	*30000*	*300*	*91*	*30*	*12*	*6*	*4*	*3*	*1.2*	*1*	*0.75*
24 h	2.11 ± 0.47	1.67 ± 0.40	1.62 ± 0.41	1.76 ± 0.24	1.94 ± 0.26	1.90 ± 0.34	2.04 ± 0.33	2.86 ± 0.36	4.72 ± 0.23	5.27 ± 0.76	6.09 ± 0.46
48 h	2.55 ± 0.52	2.40 ± 0.88	2.17 ± 0.53	2.10 ± 0.77	2.08 ± 0.53	2.55 ± 0.53	1.98 ± 0.36	2.83 ± 0.29	5.07 ± 0.41	7.70 ± 1.07	6.29 ± 0.86
72 h	2.83 ± 0.70	2.27 ± 0.31	1.96 ± 0.56	2.05 ± 0.57	2.25 ± 0.86	2.15 ± 0.67	1.90 ± 0.34	3.01 ± 0.31	6.07 ± 0.38	6.09 ± 1.67	7.05 ± 1.22
96 h	3.33 ± 0.91	2.09 ± 0.63	1.98 ± 0.36	2.38 ± 0.87	2.21 ± 0.61	2.02 ± 0.56	1.88 ± 0.23	3.99 ± 0.47	6.87 ± 0.29	7.83 ± 1.88	8.75 ± 1.44
168 h	3.40 ± 1.13	2.61 ± 0.27	2.71 ± 0.27	2.70 ± 0.28	2.68 ± 0.31	2.58 ± 0.31	2.29 ± 0.56	4.70 ± 0.37	6.66 ± 0.32	8.66 ± 1.64	8.43 ± 1.28

*The ratio of manganese(II) to copper(II) ions in each of the fermentation media is given in a separate row underneath the incrementing Cu^2+^ concentrations. Cultures were grown on D-glucose either under manganese(II) ion limitation (1.5 μg L^–1^);*

***(A)** or under manganese(II) ion sufficiency (300 μg L^–1^);*

***(B)**. All other nutrients were present at default concentrations (see “Materials and Methods” section). All results are given in micrometer (μm). The column at the left gives the sampling time in hours (h). N.G., no or negligible fungal outgrowth.*

Macro-morphology at 300 μg L^–1^ Mn^2+^ ion and low-to-medium Cu^2+^ ion concentrations was constituted by overwhelmingly loose (so-called periferal hairy) regions where pellet diameters were larger than 350 μm (Table 4B). However, in the presence of more than 250 mg L^–1^ Cu^2+^, the size of these pellets decreased to diameters of 250 μm as an average, more resembling cultures with low Mn^2+^ and Cu^2+^ (Table 4A). Essentially identical results were obtained on D-fructose as the sole carbon source at both the low and the high Mn^2+^ concentration (data not shown).

**TABLE 4A:** Average pellet size in *A. terreus* NRRL1960 D-glucose-grown cultures as a function of the copper(II) ion concentration in the culture broth at Mn(II) limitation and sufficiency.

	1.5 μg L^–^^1^ Mn^2+^										

Cu^2+^ (mg L^–1^)	0.01	1	3.3	10	25	50	75	100	250	300	400

*Mn:Cu* × *10*^3^	*150*	b	*0.45*	*0.15*	*0.06*	*0.03*	*0.02*	*0.015*	*0.006*	*0.005*	*0.00375*
24 h	250 ± 29	83 ± 13	63 ± 10	48 ± 10	46 ± 15	44 ± 10	20 ± 6	22 ± 8	20 ± 5	N.G.	N.G.
48 h	343 ± 37	86 ± 14	62 ± 15	50 ± 12	48 ± 11	40 ± 13	30 ± 10	28 ± 12	24 ± 6	N.G.	N.G.
72 h	328 ± 57	96 ± 18	68 ± 19	61 ± 21	49 ± 14	40 ± 17	37 ± 10	34 ± 10	28 ± 8	N.G.	N.G.
96 h	295 ± 48	98 ± 12	88 ± 22	65 ± 20	57 ± 13	52 ± 19	38 ± 9	37 ± 12	30 ± 9	N.G.	N.G.
168 h	233 ± 40	107 ± 11	95 ± 16	73 ± 18	67 ± 18	56 ± 16	44 ± 15	46 ± 11	35 ± 9	N.G.	N.G.

**TABLE 4B:** 

	300 μg L^–^^1^ Mn^2+^										

Cu^2+^ (mg L^–1^)	0.01	1	3.3	10	25	50	75	100	250	300	400

*Mn:Cu* × *10*^3^	*30000*	*300*	*91*	*30*	*12*	*6*	*4*	*3*	*1.2*	*1*	*0.75*
24 h	275 ± 58	167 ± 99	208 ± 86	>350	294 ± 56	290 ± 64	284 ± 63	286 ± 56	342 ± 86	212 ± 125	250 ± 63
48 h	>350	>350	>350	>350	>350	>350	>350	>350	>350	297 ± 77	233 ± 36
72 h	>350	>350	>350	>350	>350	>350	>350	>350	>350	288 ± 57	250 ± 42
96 h	>350	>350	>350	>350	>350	>350	>350	>350	>350	257 ± 58	235 ± 44
168 h	>350	>350	>350	>350	>350	>350	>350	>350	>350	226 ± 54	233 ± 48

*The ratio of manganese(II) to copper(II) in each of the fermentation media is given in a separate row underneath the incrementing Cu(II) concentrations. Cultures were grown on D-glucose as sole carbon source either under manganese(II) ion limitation (1.5 μg L^–1^);*

***(A)** or under manganese(II) ion sufficiency (300 μg L^–1^);*

***(B)**. All other nutrients were present at default concentrations (see “Materials and Methods” section). All pellet diameters are given in micrometer (μm). The column at the left gives the sampling time in hours (h). N.G., no or negligible fungal outgrowth.*

D-Xylose-grown cultures likewise displayed the typical overflow-associated morphology under manganese limitation, with average cell diameters increasing with the Cu^2+^ concentration and with the cultivation time (Table 5A). However, in contrast to what was observed on D-glucose, formation of pellets with characteristic core region was observed at lower Cu^2+^ ion concentrations tested: >350 μm pellets at 0.01 mg L^–1^ and >250 μm pellets at 1 mg L^–1^, as opposed to the morphology seen at the standard Cu^2+^ ion concentration of 3.3 mg L^–1^, optimal for itaconic acid yield also on D-xylose (Table 6A). Under Mn^2+^ ion sufficient conditions, fungal morphology on D-xylose was generally similar to that in D-glucose fermentations (Tables 5B, 6B). However, no correlation could be observed between any of the morphological parameters investigated and the itaconic acid production measured either on this pentose or on L-arabinose.

**TABLE 5A:** Average cell diameter in *A. terreus* NRRL1960 D-xylose-grown mycelia as a function of the copper(II) ion concentration in the culture broth at Mn(II) limitation and sufficiency.

	1.5 μg L^–^^1^ Mn^2+^										

Cu^2+^ (mg L^–1^)	0.01	1	3.3	10	25	50	75	100	250	300	400

*Mn:Cu* × *10*^3^	*150*	*1.5*	*0.45*	*0.15*	*0.06*	*0.03*	*0.02*	*0.015*	*0.006*	*0.005*	*0.00375*
24 h	2.15 ± 0.48	2.59 ± 1.05	3.25 ± 1.02	5.65 ± 1.21	8.18 ± 2.34	7.90 ± 2.43	7.98 ± 1.64	N.G.	N.G.	N.G.	N.G.
48 h	2.68 ± 0.49	3.01 ± 1.07	3.90 ± 1.05	5.75 ± 1.35	8.48 ± 2.65	14.53 ± 3.87	7.99 ± 1.79	N.G.	N.G.	N.G.	N.G.
72 h	2.88 ± 0.69	3.24 ± 0.98	3.96 ± 1.62	6.22 ± 2.03	8.96 ± 3.18	15.72 ± 3.08	8.54 ± 2.25	N.G.	N.G.	N.G.	N.G.
96 h	3.01 ± 0.84	3.68 ± 0.84	4.12 ± 1.19	6.30 ± 2.55	9.12 ± 2.77	16.15 ± 3.27	8.68 ± 2.95	N.G.	N.G.	N.G.	N.G.
168 h	3.22 ± 0.98	3.77 ± 0.85	4.25 ± 1.47	6.99 ± 1.98	9.31 ± 2.90	14.89 ± 3.43	9.54 ± 1.98	N.G.	N.G.	N.G.	N.G.

**TABLE 5B:** 

	300 μg L^–^^1^ Mn^2+^										

Cu^2+^ (mg L^–1^)	0.01	1	3.3	10	25	50	75	100	250	300	400

*Mn:Cu* × *10*^3^	*30000*	*300*	*91*	*30*	*12*	*6*	*4*	*3*	*1.2*	*1*	*0.75*
24 h	2.43 ± 0.58	2.92 ± 0.73	2.77 ± 0.67	2.16 ± 0.71	3.14 ± 1.06	3.90 ± 0.98	5.14 ± 0.45	4.29 ± 0.66	6.64 ± 0.99	7.25 ± 1.11	7.29 ± 0.76
48 h	3.77 ± 1.03	3.33 ± 0.59	3.72 ± 0.79	2.43 ± 0.66	3.28 ± 0.63	4.65 ± 0.73	6.89 ± 0.48	4.90 ± 1.18	6.17 ± 0.94	7.70 ± 1.17	7.49 ± 1.06
72 h	4.36 ± 0.80	3.39 ± 0.58	3.40 ± 0.95	2.25 ± 0.57	3.55 ± 0.77	5.07 ± 0.87	6.57 ± 0.98	5.10 ± 1.23	7.16 ± 0.97	7.09 ± 1.18	7.84 ± 0.99
96 h	3.80 ± 0.92	3.78 ± 0.79	2.76 ± 0.51	2.35 ± 0.54	3.84 ± 0.65	5.19 ± 0.96	5.67 ± 1.03	5.52 ± 1.78	7.97 ± 0.67	8.85 ± 1.19	8.89 ± 0.48
168 h	3.98 ± 1.03	3.66 ± 1.27	2.91 ± 0.87	2.75 ± 0.48	3.98 ± 1.30	5.78 ± 0.91	5.89 ± 0.59	5.80 ± 1.37	8.87 ± 1.00	8.91 ± 1.24	8.39 ± 1.23

*The ratio of manganese(II) to copper(II) ions in each of the fermentation media is given in a separate row underneath the incrementing Cu(II) concentrations. Cultures were grown on D-xylose as sole carbon source either under manganese(II) ion limitation (1.5 μg L^–1^);*

***(A)** or under manganese(II) ion sufficiency (300 μg L^–1^);*

***(B)**. All other nutrients were present at default concentrations (see “Materials and Methods” section). All results are given in micrometer (μm). The column at the left gives the sampling time in hours (h). N.G., no or negligible fungal outgrowth.*

**TABLE 6A:** Average pellet size in *A. terreus* NRRL1960 D-xylose-grown cultures as a function of the copper(II) ion concentration in the medium at Mn^2+^ limitation and sufficiency.

	1.5 μg L^–^^1^ Mn^2+^										

Cu^2+^ (mg L^–1^)	0.01	1	3.3	10	25	50	75	100	250	300	400

*Mn:Cu* × *10*^3^	*150*	*1.5*	*0.45*	*0.15*	*0.06*	*0.03*	*0.02*	*0.015*	*0.006*	*0.005*	*0.00375*
24 h	>350	240 ± 20	86 ± 20	68 ± 14	63 ± 17	64 ± 20	35 ± 10	N.G.	N.G.	N.G.	N.G.
48 h	>350	259 ± 34	92 ± 30	85 ± 10	78 ± 18	60 ± 18	40 ± 14	N.G.	N.G.	N.G.	N.G.
72 h	>350	265 ± 23	108 ± 25	78 ± 11	79 ± 24	69 ± 21	48 ± 11	N.G.	N.G.	N.G.	N.G.
96 h	>350	288 ± 38	106 ± 32	95 ± 18	87 ± 19	72 ± 16	48 ± 12	N.G.	N.G.	N.G.	N.G.
168 h	>350	259 ± 35	125 ± 36	98 ± 23	100 ± 20	85 ± 21	49 ± 14	N.G.	N.G.	N.G.	N.G.

**TABLE 6B:** 

	300 μg L^–^^1^ Mn^2+^										

Cu^2+^ (mg L^–1^)	0.01	1	3.3	10	25	50	75	100	250	300	400

*Mn:Cu* × *10*^3^	*30000*	*300*	*91*	*30*	*12*	*6*	*4*	*3*	*1.2*	*1*	*0.75*
24 h	258 ± 48	267 ± 99	298 ± 100	266 ± 68	312 ± 48	325 ± 49	301 ± 48	348 ± 65	350 ± 86	>350	>350
48 h	>350	>350	>350	>350	>350	>350	>350	>350	>350	>350	>350
72 h	>350	>350	>350	>350	>350	>350	>350	>350	>350	>350	>350
96 h	>350	>350	>350	>350	>350	>350	>350	>350	>350	>350	>350
168 h	>350	>350	>350	>350	>350	>350	>350	>350	>350	>350	>350

*The ratio of manganese(II) to copper(II) ions in each of the fermentation media is given in a separate row underneath the incrementing Cu^2+^ concentrations. Cultures were grown on D-xylose as sole carbon source either under manganese(II) ion limitation (1.5 μg L^–1^);*

***(A)** or under manganese(II) ion sufficiency (300 μg L^–1^);*

***(B)**. All other nutrients were present at default concentrations (see “Materials and Methods” section). All pellet diameters are given in micrometer (μm). The column at the left gives the sampling time in hours (h). N.G., no or negligible fungal outgrowth.*

## Discussion

In this paper, the effects of the interplay of Cu^2+^ and Mn^2+^ ions on growth, morphology and itaconic acid formation in *A. terreus* were analyzed and compared while growing on hexose (D-glucose, D-fructose) or pentose (D-xylose, L-arabinose) substrates, testing the hypothesis that the impact of excess Cu^2+^ ions on itaconic acid overflow is similar for all four monosaccharides. Copper is an essential transition metal ion. In fungi, it functions for instance, as a cofactor of enzymes of the respiratory chain, in free radical detoxification, pigmentation, and iron acquisition (Smith et al., 2017; Antsotegi-Uskola et al., 2020). An excess of copper ions, on the other hand, is toxic because it can inactivate other metalloenzymes by displacement of their functional divalent cation cofactor, and can catalyze the generation of radicals from hydrogen peroxide, a ubiquitous byproduct of oxidative respiration by the Fenton reaction (Smith et al., 2017; Antsotegi-Uskola et al., 2020). Cu^2+^ sensitivity is an interface between competing (micro)organisms. As an example, the innate phagocyte (alveolar macrophages and neutrophils) defense utilizes – in addition to other mechanisms – copper as a microbial toxin (Ding et al., 2014; García-Santamarina and Thiele, 2015). Consequently, all organisms have developed mechanisms to elude or alleviate Cu toxicity and intoxification (Garcia et al., 2014; Smith et al., 2017; Andrei et al., 2020; Linder, 2020). Some fungi – especially unicellular yeasts – produce copper-binding metallothioneins to modulate the concentration of the free cation (Mackie et al., 2016), whereas multicellular filamentous fungi (such as *A. fumigatus* and *Fusarium oxysporum*) make use of Cu^2+^-exporting ATPases (EC 7.2.2.9) to maintain intracellular copper homeostasis (Wiemann et al., 2017; Lorenzo-Gutiérrez et al., 2020). However, all organisms can only handle copper ions up to a certain threshold concentration, beyond which Cu-induced damage becomes irreversible.

We confirmed that *A. terreus* is sensitive to copper, and showed for four fermentable monosaccharides, that the actual toxicity threshold is dependent on the carbon growth substrate. This latter has, to the best of our knowledge, not been reported before and places a caveat on the comparisons of (heavy) metal sensitivity in the literature. Indeed, the copper tolerance is higher during growth on hexoses than on pentoses. Since the Cu^2+^-transporting ATPase is (in analogy to *A. fumigatus*; Wiemann et al., 2017) likely the major means to active copper disposal in *A. terreus* (the *A. terreus* ortholog of CrpA is encoded at locus ATET_04123; protein ID 7686^1^), our observations suggest that copper export could be facilitated by an increased ATP pool. The generation of ATP from primary carbon source catabolism is considerably different between hexoses and pentoses: the catabolism of 1 mole of D-xylose or L-arabinose leads to the formation of only 0.83 mole ATP until the entry into the tricarboxylic acid cycle (Kolláth et al., 2019), whereas the catabolism of hexoses into acetyl-CoA (glycolysis) yields 2 ATP (Supplementary Figure 3). We may therefore assume that copper export is less efficient when growing on pentoses, which may lead to toxicity at lower copper ion concentrations.

An additional (or alternative) explanation for the difference in the copper sensitivity between growth on hexoses and pentoses could be the increased requirement for NADPH reduction equivalents: as explained above, excess of copper ions generates oxidative stress (hydroxyl radicals) by catalyzing a Fenton-like reaction. Studies in *S. cerevisiae* showed that oxidative stress triggers an up-regulation of genes coding for enzymes that use specifically NADPH as a cofactor, which function to revert glutathione and thioredoxin into their reduced state (Koerkamp et al., 2002). Since the total pool of NAD(P/H) is maintained in homeostasis (Croft et al., 2020), the availability of NADPH for glutathione and thioredoxin reducing enzymes is smaller during growth on pentoses than on hexoses. It would imply that the Cu^2+^ tolerance is lower when catabolizing L-arabinose than on D-xylose, because L-arabinose catabolism requires two NADPH whereas that of D-xylose requires one (Supplementary Figure 3). This is in fact what our data have shown.

The observation that *A. terreus* tolerates higher concentrations of copper ions during conidiospore germination – in a similar carbon source dependent manner – may simply be a consequence of the short time of the germination phase when compared to the whole growth process (until carbon source exhaustion). We assume that until the end of germination only a part of the supplied copper ions have entered the cell if any. During spore germination, the cell mobilizes intracellular deposits of metabolites, including trace elements like Mn^2+^ or Cu^2+^, as the necessary uptake capacity has to be developed first.

The addition of excess copper ions to the culture medium has been patented as a method to promote high yields of citric acid during fermentations of *A. niger* on molasses (Schweiger, 1958). Subsequently, Cu^2+^ supplementation was also reported to increase itaconic acid yields in *A. terreus* (Batti and Schweiger, 1963). In these two patents, addition of copper was shown to counteract the negative effect of ferrous iron ions (Fe^2+^) on the product titers obtained. However, it was not recognized that the addition of Fe^2+^ also introduced additional Mn^2+^ ions, which are present as impurities in the iron salts utilized in the medium formulations, in sufficient amounts to account for the negative effect of Mn^2+^ on citric acid production observed in *A. niger* (Kubicek and Röhr, 1985). Kisser et al. (1980) demonstrated that Cu^2+^ ions can mitigate the inhibition of citrate accumulation in the presence of Mn^2+^ ions.

Here, we have demonstrated that Cu^2+^ ions also alleviate the negative effect of Mn^2+^ ions on itaconic acid formation in *A. terreus* (Karaffa et al., 2015; Kolláth et al., 2019), especially on hexose carbon sources. By contrast, Cu^2+^ ions could only partially counteract the Mn^2+^ inhibition on D-xylose while no alleviation could be observed in L-arabinose cultures which accumulated little itaconic acid under any cultivation regime. These differences may be related with the increased sensitivity for Cu^2+^ when the fungus is grown on pentoses.

The mechanism by which copper ions counteract the negative effect of manganese ions on itaconic (or citric-) acid formation is unclear, although it has been suggested that copper ions may compete for the manganese uptake system (Netik et al., 1997). Hockertz et al. (1987) reported that copper(II) is a potent inhibitor of a high-affinity manganese transporter system in *A. niger*, and an excess of copper would thus prevent intracellular accumulation of Mn^2+^. However, if this were the sole mechanism by which Cu^2+^ actively counteracts Mn^2+^, why does not it work on xylose?

As explained above, copper catalyzes the accumulation of oxygen radicals from hydrogen peroxide in the mitochondria which are counteracted by the activity of a manganese-dependent superoxide dismutase (MnSOD). MnSOD is usually found in the mitochondrial matrix (Weisiger and Fridovich, 1973; Okado-Matsumoto and Fridovich, 2001) and exists as a homotetramer (Ravindranath and Fridovich, 1975; Wispe et al., 1989; Borgstahl et al., 1992). Under conditions of manganese deficiency, function of MnSOD is compromised leading to severe manifestations of oxidative stress (Borrello et al., 1992; Allen et al., 2007). All metabolic changes that have been reported in *A. niger* under manganese deficiency, such as increased protein turnover and changes in plasma membrane lipid composition (Ma et al., 1981; Meixner-Monori et al., 1984) are typical for cells under oxidative stress. It is possible that – besides inhibiting manganese uptake – excess copper ions can cause increased oxidative strain by catalyzing the Fenton-like conversion of hydrogen peroxide into the highly reactive hydroxyl radical, even in the presence of manganese in concentrations that normally provide adequate protection against ROS.

We also demonstrated that interplay of Mn^2+^ and Cu^2+^ ions strongly influence the morphology of *A. terreus*. In *A. niger*, Mn^2+^-deficient morphology coincides with a significant alteration in cell wall polymer composition (Kisser et al., 1980). Mn^2+^ ions in the Golgi apparatus can influence protein glycosylation, regulation of sorting and vesicular traffic, and removal of toxic levels of ions. Mannosyltransferases located in the Golgi require Mn^2+^ as a cofactor (Lobsanov et al., 2004). Crystal structures of several glycosyltransferases revealed that Mn^2+^ binds to a conserved DXD motif in the catalytic site (Gastinel et al., 2001; Persson et al., 2001; Lobsanov et al., 2004). In *A. fumigatus*, a galactofuranosyltransferase that requires Mn^2+^, and whose deletion cripples polarized growth and the ability to sporulate has been described (Komachi et al., 2013; Katafuchi et al., 2017). A very similar phenotype has also been observed in *A. niger* during growth under manganese deficiency: galactose-containing cell wall polysaccharides (including *beta*-galactofurans and galactomannans) were strongly reduced under these conditions (Kisser et al., 1980). In analogy, the morphological changes that occur in *A. terreus* as a consequence of the availability of Mn^2+^ ions in the culture broth could be due to a requirement of a manganese cofactor by glycosyltranferases, galactofuranosyltransferases and mannosyltransferases involved in protein glycosylation and the synthesis of cell wall polysaccharides. In *A. niger*, the addition of 1 mM (=63.5 mg L^–1^) Cu^2+^ ions to cultures pregrown under manganese sufficiency results in a transition into the overflow-associated morphology typical for manganese deficiency (Kisser et al., 1980). In *A. terreus*, we observed this shift upon the addition of >300 mg L^–1^ copper, i.e., at four to five times higher concentrations, when the Mn^2+^:Cu^2+^ ratio fell below 1.2 × 10^–3^. This quantitative difference regarding manganese-copper interplay in the two *Aspergilli* remains unexplained at this time.

## Data Availability Statement

The original contributions presented in the study are included in the article/Supplementary Material, further inquiries can be directed to the corresponding author.

## Author Contributions

ES, IK, and LK conceived this study. IK, ES, EF, and LK designed the experiments. IK, VB, and ES performed the experiments. All authors analyzed the data. BK contributed essential analysis tools. LK, EF, and ES supervised the experimental work and data analysis. LK and EF wrote grant proposals and obtained funding. ES, MF, CK, and LK wrote the manuscript. All authors read and approved the final manuscript.

## Conflict of Interest

The authors declare that the research was conducted in the absence of any commercial or financial relationships that could be construed as a potential conflict of interest.

## Footnotes

1 https://mycocosm.jgi.doe.gov/Aspte1
