# Supplementary material for: Carbon-Source Dependent Interplay of Copper and Manganese Ions Modulates the Morphology and Itaconic Acid Production in Aspergillus terreus
Source: Front Microbiol. 2021 May 20;12:680420. doi: 10.3389/fmicb.2021.680420 (PMC8173074; doi:10.3389/fmicb.2021.680420)
Supplement: Supplementary file 1 [file Presentation_1.pdf]

Supplementary Material

Carbon-source dependent interplay of copper and manganese ions modulates the morphology and itaconic acid production in *Aspergillus terreus*

Erzsébet Sándor[1], István S. Kolláth[2,3], Erzsébet Fekete[2], Vivien Bíró[2,4], Michel Flipphi[2], Béla Kovács[1], Christian P. Kubicek[5], Levente Karaffa[2*]

[1]Institute of Food Science, Faculty of Agricultural and Food Science and Environmental Management, University of Debrecen, Debrecen, Hungary

[2]Department of Biochemical Engineering, Faculty of Science and Technology, University of Debrecen, Debrecen, Hungary

[3]University of Debrecen, Doctoral School of Chemistry, Debrecen, Hungary

[4]University of Debrecen, Juhász-Nagy Pál Doctoral School of Biology and Environmental Sciences, Debrecen, Hungary

[5]Institute of Chemical, Environmental & Bioscience Engineering, TU Wien, Vienna, Austria

*** Correspondence:**
Levente Karaffa

levente.karaffa@science.unideb.hu

Supplementary Figure 1: Maximal specific growth rate of *A. terreus* NRRL1960 as a function of the copper(II) ion concentration in liquid minimal media on 120 g L^{-1} D-glucose. **Panel A**: cultures grown under manganese(II) ion limitation (1.5 µg L^{-1}). **Panel B**: cultures grown under manganese(II) ion sufficiency (300 µg L^{-1}). Semi-logarithmic plots.

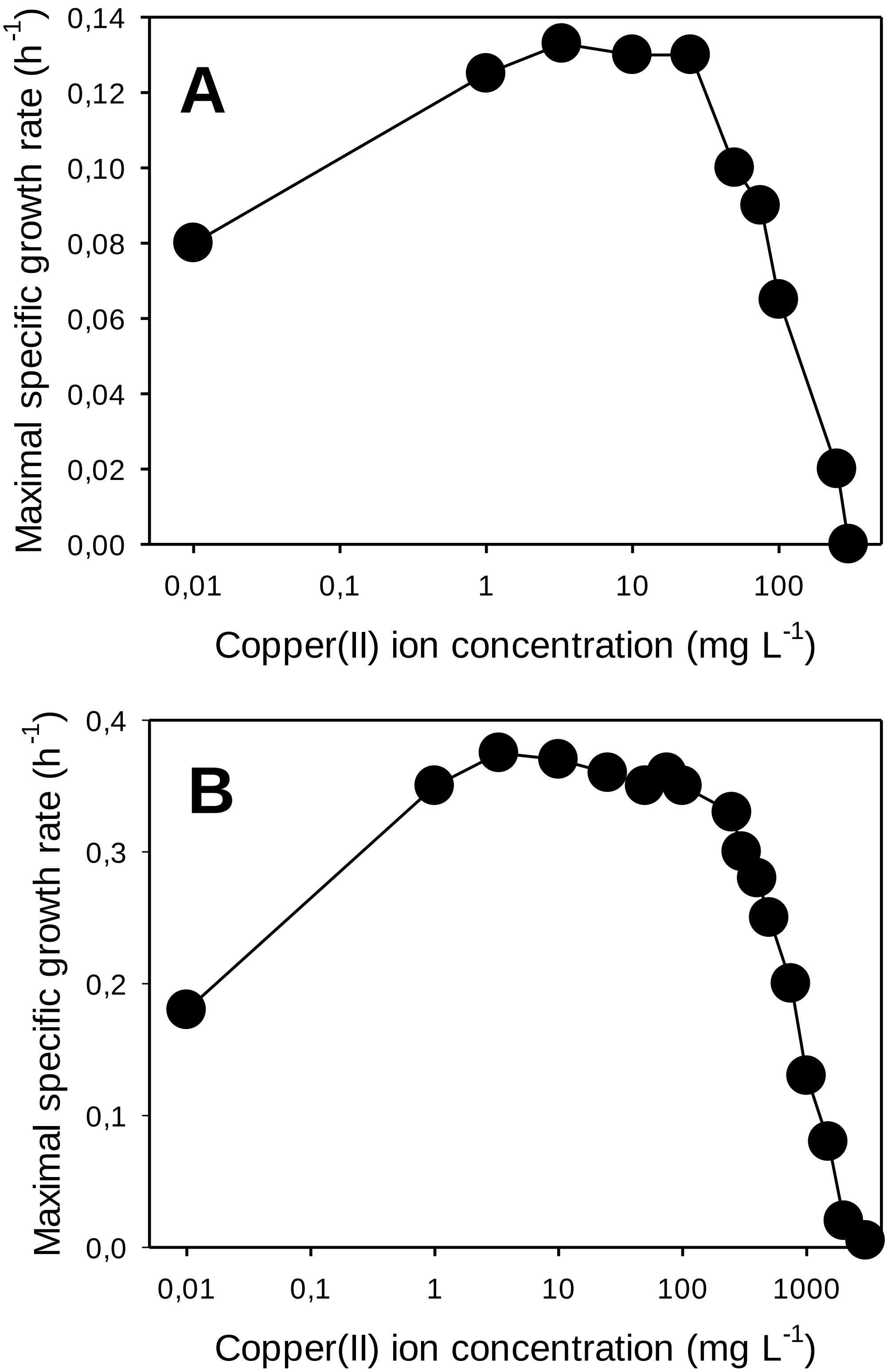
A
Maximal specific growth rate (h^{-1})
0,14
0,12
0,10
0,08
0,06
0,04
0,02
0,00
0,01
0,1
1
10
100
Copper(II) ion concentration (mg L^{-1})
B
Maximal specific growth rate (h^{-1})
0,4
0,3
0,2
0,1
0,0
0,01
0,1
1
10
100
1000
Copper(II) ion concentration (mg L^{-1})

Supplementary Figure 2: Definition of the three typical morphological forms of *Aspergillus terreus* NRRL 1960 (glucose-grown cultures). **Panel A**: "yeast-like", i.e., swollen globular cells; **Panel B**: filamentous hyphae; **Panel C**: pellet.

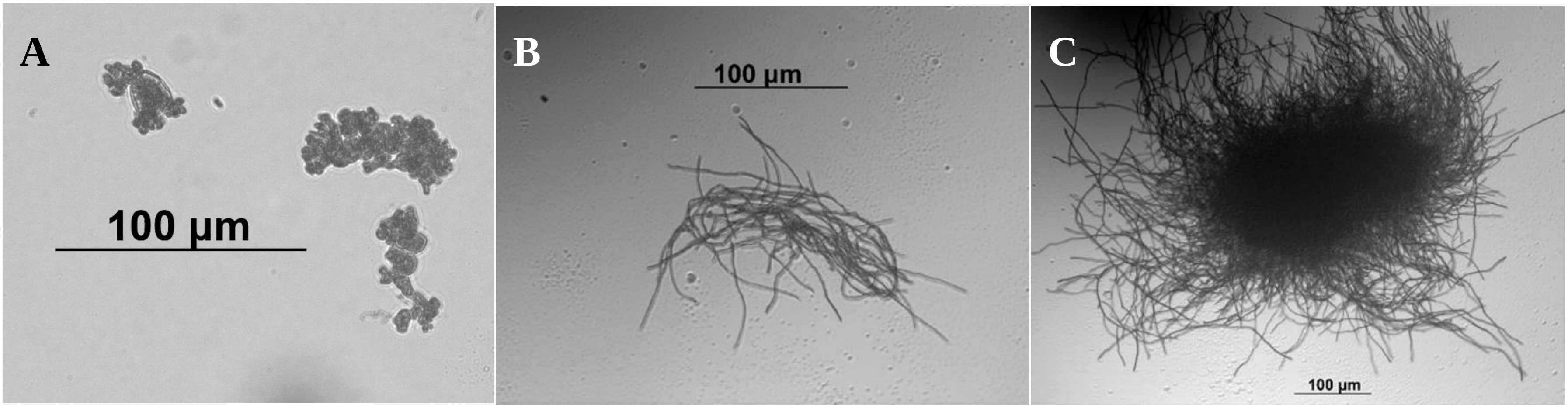

Supplementary Figure 3: Co-factor and energy balance of fungal D-xylose, L-arabinose, D-glucose and D-fructose catabolism leading to itaconic acid formation

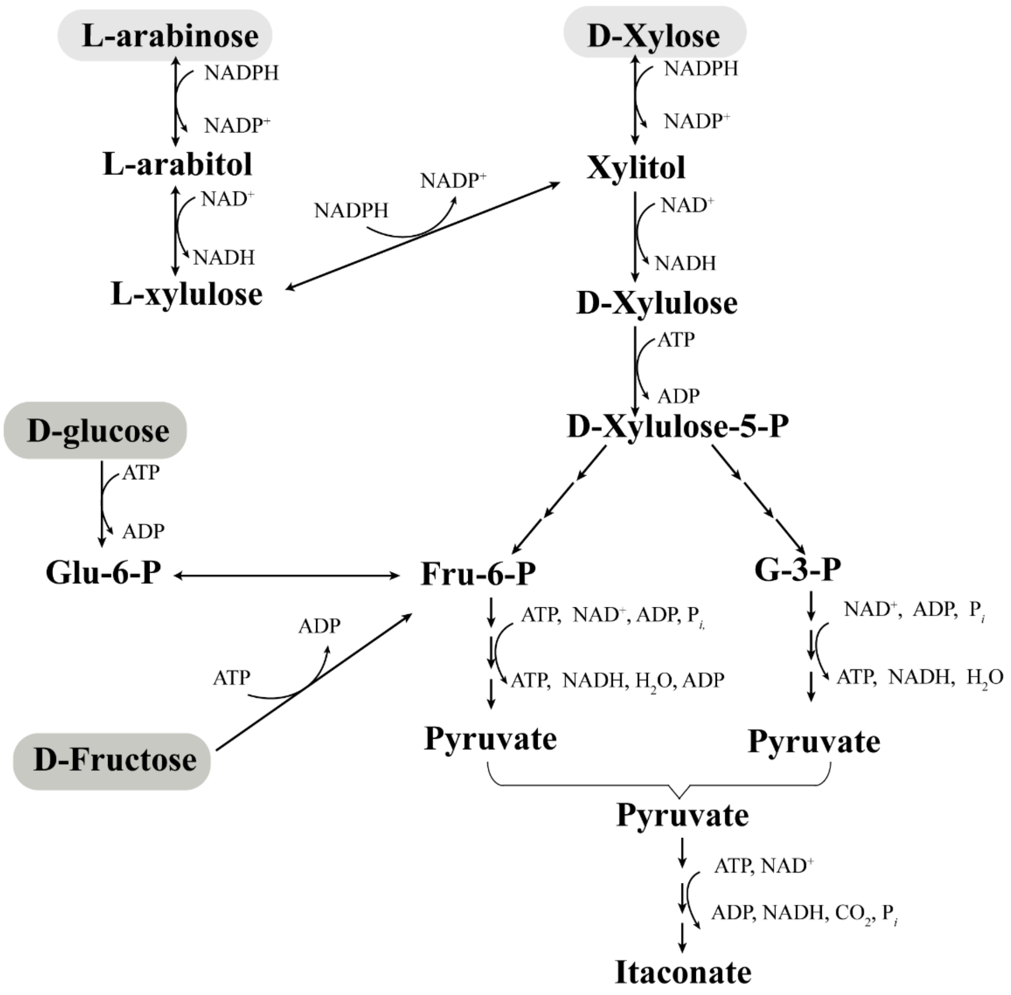

**D-Glucose + O_2 + ADP + P_i + 3 NAD^+ = ATP + 3 NADH + 2 H_2O + CO_2 + Itaconate**
**D-Fructose + O_2 + ADP + P_i + 3 NAD^+ = ATP + 3 NADH + 2 H_2O + CO_2 + Itaconate**
**D-Xylose + NADPH+ 3.5 NAD^+ + 0.83 P_i + 0.83 ADP =<br>0.83 Itaconate + $NADP^+$+ 3.5 NADH + 0.83 ATP + 0.83 H_2O + 0.83 CO_2**
**L-Arabinose + 2 NADPH+ 4.5 NAD^+ + 0.83 P_i + 0.83 ADP =<br>0.83 Itaconate + 2 $NADP^+$+ 4.5 NADH + 0.83 ATP + 0.83 H_2O + 0.83 CO_2**
